# Cellular trafficking determines the exon skipping activity of Pip6a-PMO in *mdx* skeletal and cardiac muscle cells

**DOI:** 10.1093/nar/gkt1220

**Published:** 2013-12-22

**Authors:** Taavi Lehto, Alejandra Castillo Alvarez, Sarah Gauck, Michael J. Gait, Thibault Coursindel, Matthew J. A. Wood, Bernard Lebleu, Prisca Boisguerin

**Affiliations:** ^1^UMR 5235 CNRS, Université Montpellier 2, Place Eugene Bataillon, Montpellier 34095, France, ^2^Centre de Recherche de Biochimie Macromoléculaire, UMR 5237 CNRS, 1919 Route de Mende, 34293 Montpellier, France, ^3^Universität Potsdam, Institut für Biochemie und Biologie, Maulbeerallee 2, 14469 Potsdam, Germany, ^4^Medical Research Council, Laboratory of Molecular Biology, Francis Crick Avenue, Cambridge, CB2 0QH, UK and ^5^Department of Physiology, Anatomy and Genetics, University of Oxford, South Parks Road, Oxford, OX1 3QX, UK

## Abstract

Cell-penetrating peptide-mediated delivery of phosphorodiamidate morpholino oligomers (PMOs) has shown great promise for exon-skipping therapy of Duchenne Muscular Dystrophy (DMD). Pip6a-PMO, a recently developed conjugate, is particularly efficient in a murine DMD model, although mechanisms responsible for its increased biological activity have not been studied. Here, we evaluate the cellular trafficking and the biological activity of Pip6a-PMO in skeletal muscle cells and primary cardiomyocytes. Our results indicate that Pip6a-PMO is taken up in the skeletal muscle cells by an energy- and caveolae-mediated endocytosis. Interestingly, its cellular distribution is different in undifferentiated and differentiated skeletal muscle cells (vesicular *versus* nuclear). Likewise, Pip6a-PMO mainly accumulates in cytoplasmic vesicles in primary cardiomyocytes, in which clathrin-mediated endocytosis seems to be the pre-dominant uptake pathway. These differences in cellular trafficking correspond well with the exon-skipping data, with higher activity in myotubes than in myoblasts or cardiomyocytes. These differences in cellular trafficking thus provide a possible mechanistic explanation for the variations in exon-skipping activity and restoration of dystrophin protein in heart muscle compared with skeletal muscle tissues in DMD models. Overall, Pip6a-PMO appears as the most efficient conjugate to date (low nanomolar EC_50_), even if limitations remain from endosomal escape.

## INTRODUCTION

In the past two decades, several nucleic acids-based therapeutic approaches have been investigated for the treatment of Duchenne Muscular Dystrophy (DMD) (1–3). Exon skipping via splice switching oligonucleotides (SSOs) to bypass the mutated exons of dystrophin gene has shown great promise (4–6). In this approach, SSOs target the splice sites of dystrophin pre-mRNA, induce exon skipping, enable the restoration of an open reading frame and give rise to the expression of a truncated but functional dystrophin protein (5–7). Early studies have established the viability of this strategy in animal models (mostly in *mdx* mice) of DMD (8–10). Recently, small-scale clinical trials using 2’-*O* methyl phosphorothioates (11,12) and phosphorodiamidate morpholino oligomers (PMOs) (13,14) have also established the proof-of-concept applicability of exon skipping for the potential treatment of DMD.

However, on systemic administration, naked SSOs are poorly active in inducing dystrophin restoration in skeletal muscles and are almost completely inactive even at exceptionally high doses in other important DMD-affected tissues, such as the heart (4,15–17). Clinical translation of the exon-skipping strategy will thus require the implementation of more efficient delivery strategies.

Non-viral vectors based on short peptides, called cell-penetrating peptides (CPPs), have been intensively investigated in recent years in the context of DMD (18,19). Most advances have been made with SSOs (mostly uncharged PMOs, which bind to RNA with high affinity) covalently linked to CPPs, giving rise to a series of conjugates termed peptide-PMOs or PPMOs. Initial studies were conducted with the arginine-rich (RXR)_4_ peptide (with X standing for aminohexanoic acid) (20,21) and later with its (RXRRBR)_2_ (B-peptide) derivative (22,23). Both allowed exon skipping and dystrophin rescue at much lower doses than free SSOs in skeletal muscles, with B-peptide also exhibiting some activity in the heart (24). In parallel, a R6-Penetratin (R6-Pen) conjugate (25) was proposed and further modified to improve serum stability and biological activity profile giving rise to a new class of PMO/PNA internalization peptides called Pips (26).

Further developments of these Pip peptides led to the introduction of a central hydrophobic core motif (ILFQY sequence) and generated the Pip5 series of PPMOs (27). These PPMOs were extensively screened in the *mdx* mouse model of DMD and Pip5e-PMO was identified as a promising candidate that allowed high-dystrophin restoration in both skeletal and cardiac muscles, although with lower efficiency in the latter ones (27). Additional structure-activity studies, using Pip5e as a starting sequence, were carried out to identify which elements in this peptide are important for improving cardiac delivery. These novel derivatives were named Pip6 peptides (28). The data showed that heart muscle activity was due to the presence of a central 5-amino acid hydrophobic sequence, but the exact sequence seemed less important than its length. Among these PPMOs belonging to the Pip6 family, Pip6a-PMO has been chosen for further studies such as pharmacokinetics, biodistribution and muscle physiology. However, little is known to date concerning the cellular trafficking of PMOs and PPMOs, such as Pip6a-PMO, in different muscle cell types. High doses of naked PMO do allow efficient delivery to the DMD-affected skeletal muscles *in vivo* (29). Unaided cellular uptake of PMO is thought to be dependent on the increased sarcolemmal membrane permeability, as lack of dystrophin renders the cellular membrane prone to mechanical stress-induced disruptions, making them leaky (30). However, to our knowledge, there is no direct evidence supporting this hypothesis. It is also unknown if there are major differences between the uptake of PMOs and PPMOs into undifferentiated and differentiated skeletal muscle cells, i.e. myoblasts and myotubes, that would be responsible for the often uneven restoration of dystrophin in different muscle fibres. Furthermore, heart muscle always seems to represent a more challenging barrier to achieve efficient dystrophin correction, as naked PMOs are only weakly active at high repeated doses in this tissue (29), whereas PPMO conjugates are more active but give less activity in heart than in skeletal muscle (27,28).

In this study, we have compared the exon-skipping activity and the cellular trafficking of Pip6a-PMO in relevant muscular cells, namely, a H2k *mdx* cell line (as representative of skeletal muscle), in primary cardiomyocytes isolated from newborn *mdx* mice, as well as in wild-type skeletal and cardiac muscle cells.

## MATERIALS AND METHODS

### Synthesis of peptide-PMO conjugates

Pip6a (Ac-RXRRBRRXRYQFLIRXRBRXRB-OH, with B = β-alanine and X = amino hexanoic acid) was synthesized by standard Fmoc chemistry as a C-terminal carboxylic acid and purified by high-performance liquid chromatography (27). PMO (5′-GGCCAAACCTCGGCTTACCTGAAAT-3′) was purchased from Gene Tools LLC (Corvallis, OR) with a 5′-amino linker and a 3′-fluorescein label. Pip6a was conjugated to the PMO through an amide linkage via the 5′-amino linker as described in (28). Pip6a-PMO carries the 3′-fluorescein label unless specifically mentioned in Figures as unlabelled. Unlabelled Pip6a-PMO was synthesized as previously described (27).

### Cell culture

Murine H2k *mdx* myoblasts were cultured in gelatin (0.01%)-coated flasks at 33°C, under 10% CO_2_, in Dulbecco’s modified Eagles medium (DMEM PAA laboratories) supplemented with 20% heat-inactivated fetal bovine serum (FBS Gold, PAA laboratories), 2% chicken embryo extract (Seralab), 1% penicillin-streptomycin-neomycin antibiotic mixture (PSN, Gibco) and 3 pg/µl γ-interferon (PeproTech).Murine C2C12 myoblasts (ATCC: CRL–1772™) were grown in DMEM supplemented with 10% heat inactivated FBS (PAA laboratories) and 1% PSN.

Cells were seeded in gelatin (0.01%)-coated 24-well plates at a density of 4 × 10^5^ cell/ml and left for 2 days at 33°C, 10% CO_2_, before being used as undifferentiated myoblasts. To differentiate into myotubes, cells were further grown in DMEM supplemented with 5% horse serum (Sigma-Aldrich) and 1% PSN at 37°C, 5% CO_2_ for 5 days for the H2k *mdx* and 6 days for the C2C12 cells. The differentiation of H2k *mdx* and C2C12 cells were monitored by measuring the Troponin T level by ‘western blot’ (data not shown).

Primary cardiomyocytes were isolated from the ventricles of 1- to 2-day-old newborn wild-type C57BL/6 J mice (Charles River) or *mdx* mice (provided by M. Wood) by enzymatic digestion with type-4 collagenase (Serlabo Technologies) and pancreatin (Sigma-Aldrich), as described previously (31). Briefly, freshly isolated cells were seeded in T25 flasks to allow selective adhesion of cardiac fibroblasts (32) in plating medium: 250 ml DMEM (PAA laboratories), 250 ml M199 (Sigma-Aldrich), 5 ml glutamax (100X, Gibco®, Life Technologies), 5 ml PS, 50 ml horse serum (HS, Sigma-Aldrich) and 25 ml FBS (PAA laboratories). Cardiomyocytes remaining in the supernatant were seeded in well culture plates coated with 0.01% gelatin (Sigma-Aldrich). Cardiomyocytes were used after 2–3 days when beating.

### Cell transfection

In all, 4 × 10^4^ cells/ml of H2k *mdx* or C2C12 myoblasts were seeded on gelatin (0.01%)-coated 12-well plates and grown for 2 days (myoblasts) or differentiated to myotubes as mentioned earlier in text. The 8 × 10^4^ cells/ml of cardiomyocytes were seeded on gelatin (0.01%)-coated 24 well-plates and grown until beating was observed (2–3 days). Thereafter, cells were incubated with 3′-fluorescein-labelled Pip6a-PMO, unlabelled Pip6a-PMO or 3′-fluorescein labelled PMO at the indicated concentrations. Transfections were carried out in a final volume of 500 (12-well plate) or 2000 µl (microscopy) for 4 h in OptiMEM or complete medium. For the 24-h post-treatment conditions, 1000 µl complete medium was added to each well.

### Fluorescence spectroscopy

After incubation with 3′-fluorescein labelled Pip6a-PMO or 3′-fluorescein-labelled PMO, cells were washed twice with PBS and lysed with 300 µl GLB buffer (Promega). In all, 100 µl of the lysate were transferred into black non-binding 96-well plates (Greiner) and fluorescence was measured on a Tecan plate reader (Ex 485 nm/Em 520 nm). Protein concentration was measured by a BCA kit (Pierce) on a Tecan plate reader (Abs 560 nm).

### RNA extraction

Total RNA was extracted using a Nucleospin kit (Macherey-Nagel) from each single well for the 24 h treatment or by Trizol extraction (TRI Reagent® RNA Isolation Reagent, Sigma-Aldrich) by pooling duplicates for the 4 h treatment as per manufacturers’ instructions. Total RNA quantification was done using Nanodrop® ND-1000 (Thermo Scientific).

### RT-PCR and nested PCR

The 400 ng of RNA template was used in a 2-step RT-PCR reaction using GeneAMP RNA PCR kit (Invitrogen Life Technology) with gene-specific primers (Exon20Fo 5′-CAG AAT TCT GCC AAT TGC TGAG-3′ and Exon26Ro 5′-TTC TTC AGC TTG TGT CAT CC-3′). Cycle conditions were as follows: first step: 42°C for 30 min, 94°C for 15 min and 5°C for 5 min;. second step: 95°C for 2 min, followed by 30 cycles of 95°C for 30 s, 58°C for 1 min, 72°C for 2 min and a final extension at 72°C for 10 min. Two microliters of the RT-PCR product was used as a template in secondary nested PCR using Amplitaq Gold DNA polymerase kit (Invitrogen Life Technology) with gene-specific primers (Exon20Fi 5′-CCC AGT CTA CCA CCC TAT CAG AGC-3′ and Exon26Ri 5′-CCT GCC TTT AAG GCT TCC TT-3′). Cycle conditions were as follows: 95°C for 10 min followed by 22 cycles of 95°C for 30 s, 58°C for 1 min, 72°C for 2 min and a final extension at 72°C for 10 min. Products were fractionated by electrophoresis in 2.5% agarose gels (100 V for 1 h) and quantified for percentage of exon skipping (%ES) by densitometric analysis (BioRad). %ES is calculated on the densitometric value of the Δ23 band related to the other bands (full length + Δ23 + Δ23 + 22).

### Endocytosis inhibition assays

In all, 4 × 10^4^ cells/ml were seeded on gelatin (0.01%)-coated 24-well plates and grown for 2 days before differentiation as mentioned earlier in text. Cells were pre-treated for 30 min with inhibitors (all provided from Sigma-Aldrich): 10 mM NaN_3_ and 6 mM 2′-Deoxy-D-Glucose (DDG) for ATP-depletion (-ATP); 30 or 15 µM chlorpromazine (CPZ) for clathrin-mediated endocytosis inhibition; 50 or 25 µM nystatin (Nys), 150 µM or 200 µM genistein, 2.5 or 4 µg/ml filipin III for caveolae-mediated endocytosis inhibition; and 10 µM 5-(N-ethyl-N-isopropyl) amilorid (EIPA) for macropinocytosis inhibition. Thereafter, cells were treated with Pip6a-PMO for 4 h in the presence of the inhibitors. After washing twice with PBS, cells were lysed with GLB buffer (Promega) and cell lysates were evaluated by fluorescence spectroscopy and RT-PCR as described earlier in text.

### Fluorescence microscopy

The 2 × 10^5^ cells/dish of H2k *mdx* or C2C12 were seeded on gelatin (0.01%)-coated dishes and grown for 2 days (myoblasts) or were differentiated as mention earlier in text. In all, 3 × 10^5^ cells/dish of cardiomyocytes were seeded on gelatin (0.01%)-coated dishes and grown until cells were beating (2–3 days). Cells were then transfected with Pip6a-PMO-CF (1 µM) or PMO-CF (1 µM) for 1 h or 4 h. Ten minutes before the end of the incubation, 1 µl Hoechst 33342 dye (Invitrogen Life technology) was added to each dish to visualize the nucleus. The excess of conjugate or dye was eliminated by washing (2x PBS). Finally, 2 ml of OptiMEM were added as a final volume and 10 µl of trypan blue solution (Invitrogen Life technology) were added to quench extracellular fluorescence. Fluorescent images were captured immediately using a Zeiss Axiovert 200 M fluorescence microscope and the Axio Vision software (Carl Zeiss).

### Statistical analysis

All values are expressed as means±SEM. Multiple comparisons between groups were assessed by one-way ANOVA with Newman–Keuls post hoc test or T test when appropriate. *P* values were noted as: ** for *P*<0.001 and *** for *P*<0.0001.

Data were analyzed using GraphPad Prism GraphPad Software, San Diego California, USA.

## RESULTS

### Pip6a-PMO-mediated exon skipping in *mdx* skeletal muscle cells

We first aimed to confirm the potential of Pip6a-PMO to induce exon 23 skipping in the well-established skeletal muscle model of DMD, i.e. murine H2k *mdx* cells (18). To track Pip6a-PMO in the intracellular compartments, the PMO part of the conjugate was 3′-labelled with fluorescein and this compound was used throughout all experiments (if not indicated otherwise).

**Figure 1.**
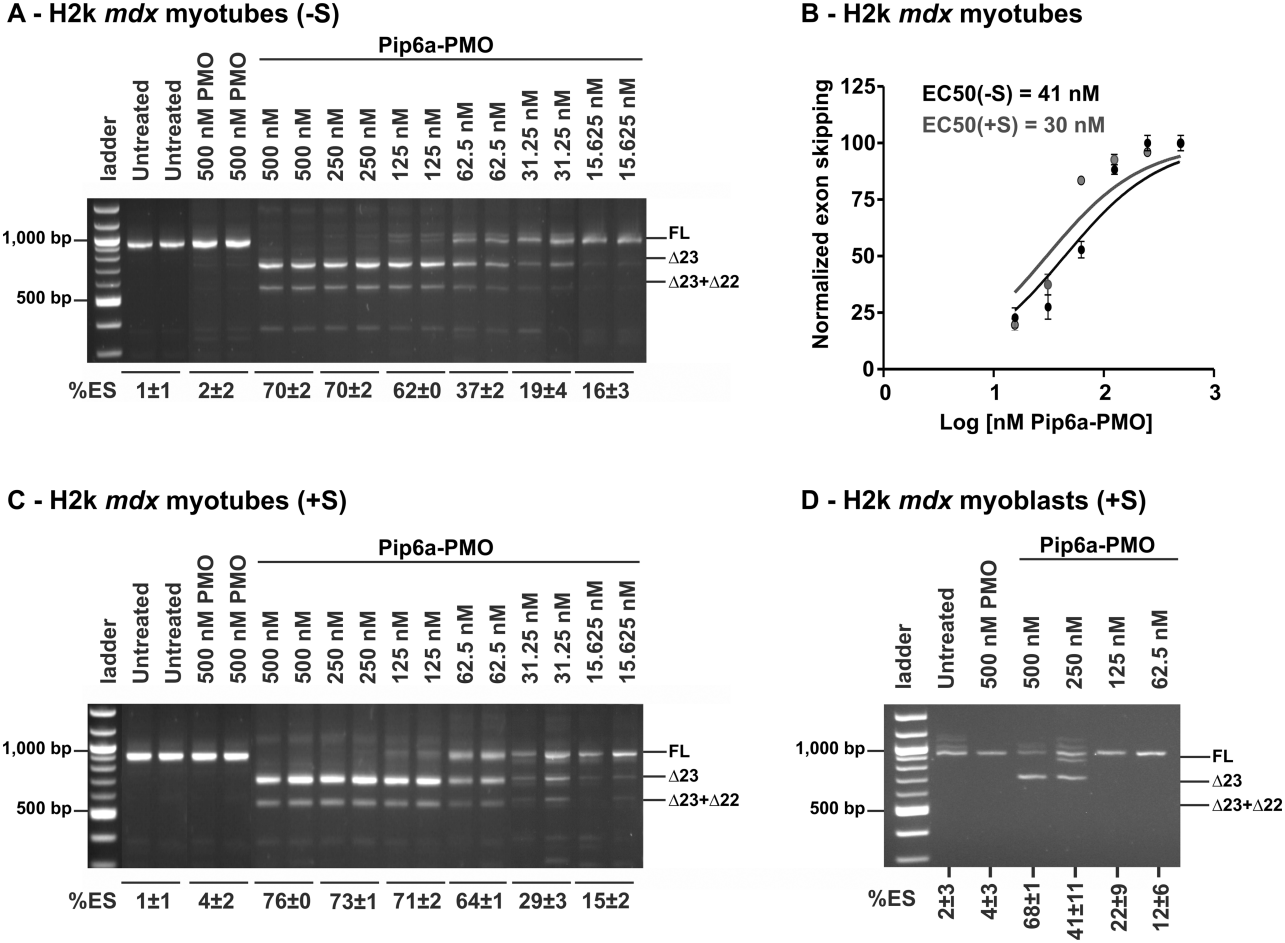
Exon-skipping efficiency of Pip6a-PMO in *mdx* skeletal muscle cells. H2k *mdx* cells were differentiated for 5 days and thereafter incubated with Pip6a-PMO under serum-free (–S) (**A**) or serum conditions (+S) (**C**). Graphical view and calculation of half-maximal effective concentration (EC_50_) at 24 h post-treatment for exon skipping under serum-free and serum condition (**B**). EC50 values were calculated using Prism 5.0 software after normalization of the Δ23 skipping. Pip6a-PMO-meditated exon skipping in H2k *mdx* myoblasts incubated under serum conditions (+S) (**D**). Transfections were carried out for 4 h under serum-free or serum condition and cells were incubated further with serum-containing medium for 20 h. Exon-skipping efficiency was evaluated on pre-mRNA levels by nested RT-PCR. Products were separated by gel electrophoresis and exon-skipping values were derived from densitometric analysis. For each experiment, percentage of exon skipping (%ES) is calculated on the densitometric value of the Δ23 band related to the other bands (full length + Δ23 + Δ23 + 22) with *n* ≥ 4.

*In vitro* differentiated H2k *mdx* myotubes (as verified by their morphology and by the expression of the troponin T marker) were treated with increasing concentrations of Pip6a-PMO, ranging from 15 to 500 nM, and exon skipping was evaluated after 24 h treatment by nested RT-PCR. In line with a previous report (28), Pip6a-PMO induced high levels of exon skipping in a dose-dependent manner (Figure 1A), reaching full skipping at 250 nM. An EC_50_ value (i.e. concentration required to achieve 50% exon 23 skipping) of 41 nM was calculated (Figure 1B) when transfections were carried out in serum-free medium (-S).

The delivery potential of most CPP-based vectors is often negatively influenced by the presence of serum proteins. To explore the impact of serum proteins on Pip6a-PMO transfection, experiments were carried out in parallel in serum-supplemented transfection medium (+S). The presence of serum did not have any impact on Pip6a-PMO’s exon-skipping activity (Figure 1C) and an even slightly lower EC_50_ value of 30 nM was registered (Figure 1B).

Next, we evaluated exon-skipping activity in undifferentiated H2k *mdx* myoblasts and found it much lower than for myotubes as, even at 500 nM, Pip6a-PMO was unable to induce full exon 23 skipping (Figure 1D). An EC50 of 183 nM was recorded for Pip6a-PMO in H2k *mdx* myoblasts (data not shown), which is ∼6-times lower than in the H2k *mdx* myotubes.

We also monitored exon-23 skipping 48 h post-treatment and a more pronounced exon-skipping activity was achieved compared with 24 h treatment (EC_50_ at 48 h = 20 nM) (Supplementary Figure S1). We also verified that the unlabelled Pip6a-PMO conjugate had a similar biological activity profile as compared with the fluorescently labelled conjugate (Supplementary Figure S2).

Taken together, these studies show that Pip6a-PMO enables efficient exon skipping at low concentrations (as indicated by EC_50_ values) and is completely resistant to interference by serum proteins in line with its ability to restore efficiently dystrophin expression on systemic administration in *mdx* mice (28).

### Uptake and exon-skipping efficiency of Pip6a-PMO in normal C2C12 and H2k *mdx* skeletal muscle cells

DMD-affected skeletal muscles have been reported as being more permeable than healthy muscles and increased leakiness has been proposed as a possible explanation for the activity of naked SSOs in animal models and in clinical trials [reviewed in (19)]. We have used *in vitro* differentiated C2C12 cells as a model of normal skeletal muscle cells and evaluated exon-skipping efficiency in comparison with Hk2 *mdx* myotubes. As shown in Supplementary Figure S3, exon-skipping efficiency measured 24 h post-treatment was slightly lower in differentiated C2C12 myotubes (with an EC_50_ of 107 nM using serum-containing transfection media), as compared with H2k *mdx* myotubes (Figure 1B and C).

**Figure 2.**
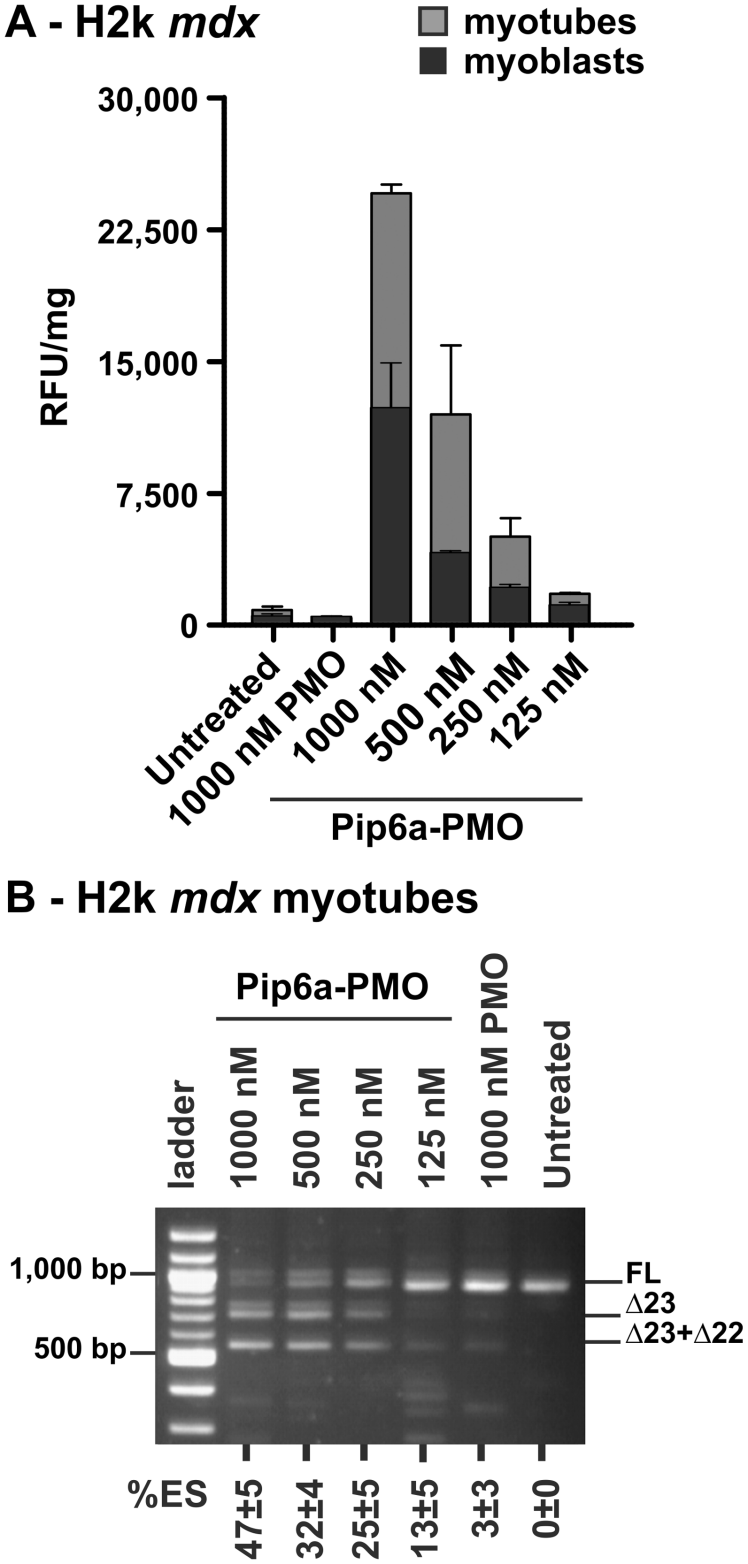
Comparative analysis of Pip6a-PMO uptake and exon skipping in H2k *mdx* skeletal muscle cells. Differentiated and non-differentiated H2k *mdx* cells were incubated 4 h with Pip6a-PMO at the indicated concentrations. The dose-depended internalization was assessed by fluorescence spectroscopy (**A**). Using the same differentiated H2K *mdx* samples, exon-skipping efficiency cells was evaluated by RT-PCR (**B**). (%ES calculated as described in Figure 1 with *n* ≥ 4).

As our main goal was to elucidate Pip6a-PMO cellular trafficking, we established and optimized experimental conditions allowing cellular uptake and exon 23-skipping activity measurements within the same samples. A 4 h post-treatment time point was found optimal to generate a strong fluorescence signal (Supplementary Figure S4) and to allow exon 23-skipping detection. Differentiated mouse H2k *mdx* and C2C12 myotubes were treated with increasing Pip6a-PMO concentrations, ranging from 125–1000 nM. Cellular uptake was monitored by fluorescence spectroscopy, as FACS analysis cannot be performed with myotubes. Pip6a-PMO entered the cells in a dose-dependent manner in both H2k *mdx* and C2C12 myotubes, although no uptake of naked PMO could be detected even at 1000 nM (Figure 2A). Minimal differences in the uptake profile of Pip6a-PMO in H2k *mdx* (Figure 2A) and C2C12 (Supplementary Figure S5A) myotubes were observed, although fluorescence levels were slightly more elevated in H2k myotubes. We then evaluated exon 23-skipping within the same samples by nested RT-PCR. Dose-dependent uptake with Pip6a-PMO corroborated well with exon-skipping levels in both H2k *mdx* (Figure 2B) and C2C12 (Supplementary Figure S5B). No significant difference in exon skipping was found between H2k *mdx* and C2C12 myotubes at 4 h post-treatment, in line with the uptake data.

### Cellular trafficking of Pip6a-PMO in skeletal muscle myoblasts and myotubes

Reaching efficiently the cell cytosol or nucleus is considered a major hurdle in the CPP-mediated delivery of oligonucleotides (33,34). In most cases, CPPs are efficiently taken up, but remain segregated in endocytic vesicles. As the intracellular fate of CPPs and their cargo is known to vary highly with different cell lines and physiological conditions, we have studied the uptake and intracellular distribution of Pip6a-PMO in undifferentiated myoblasts and in differentiated myotubes of H2k *mdx* and C2C12 cell lines.

Myoblasts and myotubes were treated in the same conditions with varying concentrations of Pip6a-PMO and uptake was measured by fluorescence spectroscopy. Pip6a-PMO was more efficiently taken up by H2k *mdx* myotubes than by H2k *mdx* myoblasts (Figure 2A), in line with exon-skipping activity (Figure 1). A similar trend was observed in normal C2C12 myoblasts and myotubes (Supplementary Figure S5A). At the same time, naked PMOs were not taken up to any detectable levels under identical conditions (Figure 2 and Supplementary Figure S5). These data indicate that internalization of Pip6a-PMO clearly differs between the undifferentiated myoblasts and differentiated myotubes. However, this difference in cellular trafficking and exon-skipping activity is not due to a higher nuclei density in myotubes. Using the same fluorescence microscopy picture, no significant differences between both conditions were found with 19 ± 5 and 25 ± 5 nuclei per microscopic image in myoblasts and myotubes, respectively (with *n* > 8, data not shown).

**Figure 3.**
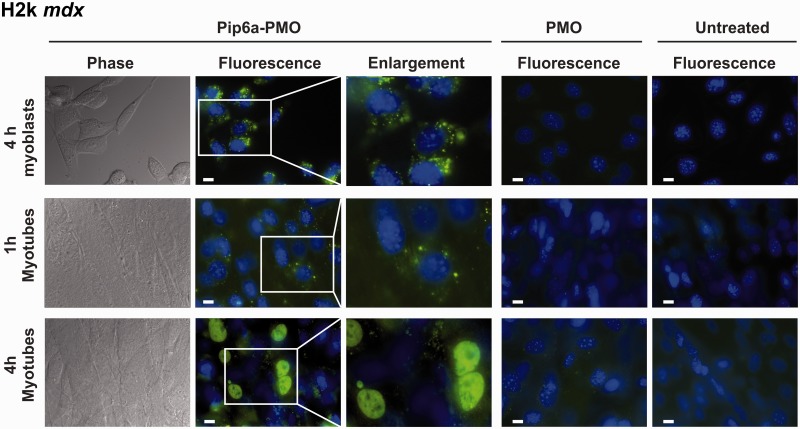
Intracellular distribution of Pip6a-PMO in H2k *mdx* myoblasts and myotubes. Representative images of H2K *mdx* myoblasts or myotubes which were incubated with fluorescein-labelled Pip6a-PMO (1000 nM) or PMO (1000 nM) for the indicated times. Cell nuclei were labelled with Hoechst dye. Untreated cells are shown as controls. Pip6a-PMO or naked PMO distribution in live unfixed cells was evaluated by fluorescence microscopy with a Zeiss Axiovert 200 M. White bar = 10 µm.

We next evaluated whether differences in cellular trafficking between myoblasts and myotubes of both H2k *mdx* and C2C12 cells could also be seen. H2k *mdx* myoblasts and myotubes were treated with Pip6a-PMO, and its cellular distribution was analysed by fluorescence microscopy. Strikingly, the intracellular distribution of Pip6a-PMO was rather different in H2k *mdx* myoblasts and myotubes (Figure 3). In myoblasts, Pip6a-PMO had a mostly cytoplasmic punctate distribution pattern reminiscent of association with endocytic vesicles and little fluorescence arose from cell nuclei. Longer incubation periods (6 h or 8 h), as well as 4 h post-treatment time-lapse experiments (12 h) clearly demonstrated that this dotted Pip6a-PMO pattern did not change over time (data not shown).

Nearly the same internalization pattern was observed in H2k *mdx* myotubes after a short (1 h) incubation (Figure 3). On the contrary, at longer incubation times (4 h), Pip6a-PMO was diffusely distributed in the cytoplasm of myotubes and a strong fluorescent signal was recorded in the nuclei (Figure 3). Similar patterns were seen in C2C12 myoblasts and myotubes (Supplementary Figure S5C). Treatments with naked PMO did not yield any detectable fluorescent signal in all analysed conditions (myoblasts and myotubes of H2k *mdx* and C2C12 cells) (Figure 3 and Supplementary Figure S5C).

These results indicate strong differences in the intracellular trafficking of Pip6a-PMO in differentiated and undifferentiated skeletal muscle cells. We observed a 2-fold increased uptake in myotubes (Figure 2) and, importantly, a stronger nuclear accumulation in myotubes than in myoblasts.

### Elucidating the cellular trafficking of Pip6a-PMO in *mdx* skeletal muscle cells

**Figure 4.**
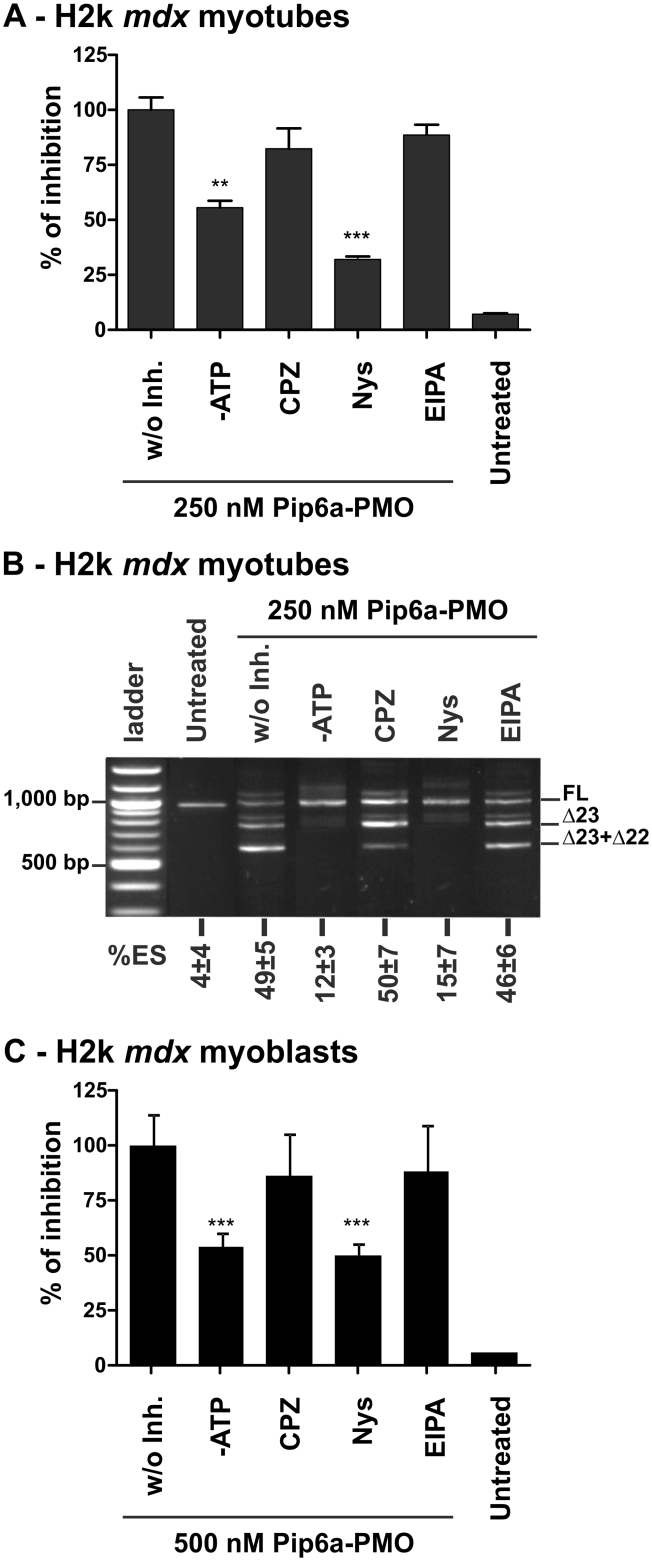
Cellular trafficking of Pip6a-PMO in *mdx* skeletal muscle cells. Effect of energy depletion and endocytosis inhibitors on the uptake of Pip6a-PMO (at 250 nM) in H2k *mdx* myotubes at 4 h post-treatment as measured by fluorescence spectroscopy (*n* ≥ 4) (**A**) with the corresponding exon skipping activity of the same samples (**B**). (%ES calculated as described in Figure 1). Treatment with inhibitors was started 30 min before the treatment with Pip6a-PMO. The following inhibitors were used at indicated concentrations: NaN_3_ (10 mM) and 2′-Deoxy-D-Glucose (DDG) (6 mM) for ATP-depletion (-ATP); chlorpromazine (CPZ, 30 µM) for clathrin-mediated endocytosis inhibition; nystatin (Nys, 50 µM) for caveolae-mediated endocytosis inhibition; and 5-(N-ethyl-N-isopropyl) amiloride (EIPA, 10 µM) for macropinocytosis inhibition (*n* ≥ 4). (**C**) Effect of energy depletion and endocytosis inhibitors on the uptake of Pip6a-PMO (at 500 nM) in H2k *mdx* myoblasts at 4 h post-treatment as measured by fluorescence spectroscopy. For myoblasts chlorpromazine (CPZ) and nystatin (Nys) were used at 15 µM and 25 µM, respectively (*n* ≥ 4).

CPPs are generally believed to use different routes of endoytosis to gain access to cells, especially when attached to cargo molecules (35). To determine which endocytotic pathways are involved in the trafficking of Pip6a-PMO in the H2k *mdx* myotubes, we took advantage of pharmacological inhibitors of the major endocytic pathways. Differentiated H2k *mdx* myotubes were pre-treated with the inhibitors for 30 min—NaN_3_ and 2′-Deoxy-D-Glucose (DDG) for ATP-depletion (-ATP); chlorpromazine (CPZ) for clathrin-mediated endocytosis inhibition; nystatin (Nys) for caveolae-mediated endocytosis inhibition; and 5-(*N*-ethyl-*N*-isopropyl) amiloride (EIPA) for macropinocytosis inhibition. Cells were then treated with Pip6a-PMO at a 250 nM concentration for 4 h in the presence of the inhibitors. Fluorescence spectroscopy analysis showed that energy-depletion significantly decreased the uptake of Pip6a-PMO (Figure 4A), although the exon-skipping activity was completely abolished (Figure 4B). Similar effects were recorded when the experiments were carried out at 4°C under conditions where energy-dependent processes are greatly slowed down (data not shown).

From the pharmacological inhibitors of endocytosis only nystatin, which is specific of caveolae-mediated endocytosis, had a pronounced effect on both uptake (Figure 4A) and exon 23-skipping efficiency (Figure 4B) of Pip6a-PMO. The involvement of caveolae-mediated endocytosis was confirmed with additional caveolae inhibitors, such as Filipin III and Genistein that also inhibited both uptake and exon skiping activity of Pip6a-PMO (Supplementary Figure S6). CPZ and EIPA, inhibitors of clathrin-mediated endocytosis and macropinocytosis, respectively, had only negligible effects on cellular delivery and exon-skipping activity (Figure 4).

Similar data were obtained in undifferentiated H2k *mdx* myoblasts (Figure 4C), indicating that the observed differences in the intracellular distribution between myoblasts and myotubes could not arise from the use of different uptake pathways. Unfortunately, under the same condition (24-well plate, 500 µl incubation volume) as used for myotube analysis, we were unable to extract enough RNA for a representative band detection in myoblasts (data not shown).

Altogether, these results strongly suggest that energy-dependent processes involving caveolae-mediated endocytosis are mainly responsible for the uptake of Pip6a-PMO in H2k *mdx* cells.

### Cellular trafficking and exon-skipping efficiency of Pip6a-PMO in cardiac muscle cells

Inducing biological activity in heart muscle remains a substantial obstacle for SSO-based therapy for DMD, as observed with naked PMO. Conjugation to CPP carriers enables improved dystrophin rescue in the cardiac tissue albeit to a much lower level when compared with dystrophin restoration in skeletal muscles. This stands also for Pip6a, even-though it is by far the most active SSO delivery vector for the heart. To our knowledge, investigations of barriers encountered in the heart muscle cells that might impede dystrophin rescue have received little attention. To have an insight to this, we have studied in parallel the cellular trafficking and the exon-skipping activity of Pip6a-PMO in primary cardiomyocytes derived from neonatal *mdx* or wild-type mice hearts.

**Figure 5.**
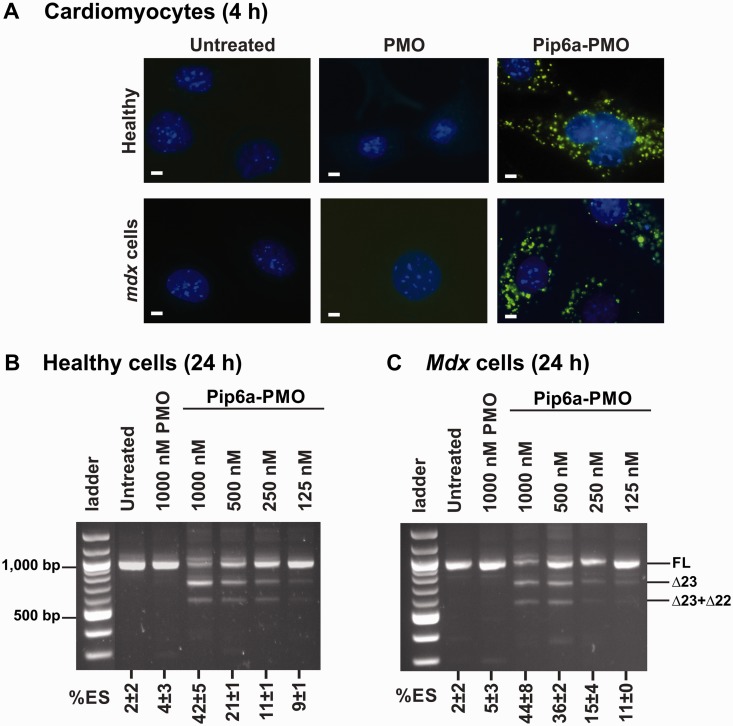
Cellular trafficking and exon-skipping efficiency of Pip6a-PMO in heart muscle cells. (**A**) Representative images of the cellular distribution of Pip6a-PMO in primary wild-type (WT) and *mdx* cardiomyocytes at 4 h as measured by fluorescence microscopy (Pip6a-PMO and PMO used at 1000 nM). Cell nuclei were labelled with Hoechst dye. Untreated cells are shown as controls. White bar = 10 µm. Exon-skipping efficiency of Pip6a-PMO in serum-containing medium at 24 h post-treatment in primary WT (**B**) and *mdx* (**C**) cardiomyocytes (*n* ≥ 4). RT-PCR analysis and %ES calculation as described in Figure 1.

Primary *mdx* and wild-type cardiomyocytes were treated with Pip6a-PMO for 4 h and intracellular distribution was analysed by fluorescence microscopy. As indicated by the punctuated distribution pattern and the faint nuclear signal, Pip6a-PMO remains largely segregated in endocytic vesicles in these fully differentiated cells (Figure 5A, right panel). This type of distribution pattern is similar to the one seen in undifferentiated H2k *mdx* or C2C12 myoblasts. We also confirmed limited or no signal from the cells treated with PMO (Figure 5A, middle panel) or untreated (Figure 5A, left panel) controls.

We next investigated the exon-skipping activity of Pip6a-PMO in these *mdx* and wild-type cardiac muscle cells. Normal and *mdx* cardiomyocytes were treated with Pip6a-PMO at various concentrations, ranging from 125 to 1000 nM, and exon 23 skipping was evaluated 24 h post-treatment. Pip6a-PMO induced a dose-dependent exon skipping in normal cardiomyocytes, rising up to 44% at 1000 nM of Pip6a-PMO. In *mdx* cardiomyocytes, similar effects were seen, with exon-skipping efficiency rising from ∼10% exon 23 skipping at 250 nM to ∼40% at 1000 nM (Figure 5B), thus reaching significantly lower values than in skeletal muscle cells. In control experiments, naked PMO at 1000 nM concentration failed to induce any exon skipping in cardiomyocytes (Figure 5B and C). At variance with H2k *mdx* myotubes, exon skipping did not increase significantly with 48 h incubations in cardiomyocytes (Supplementary Figure S7).

**Figure 6.**
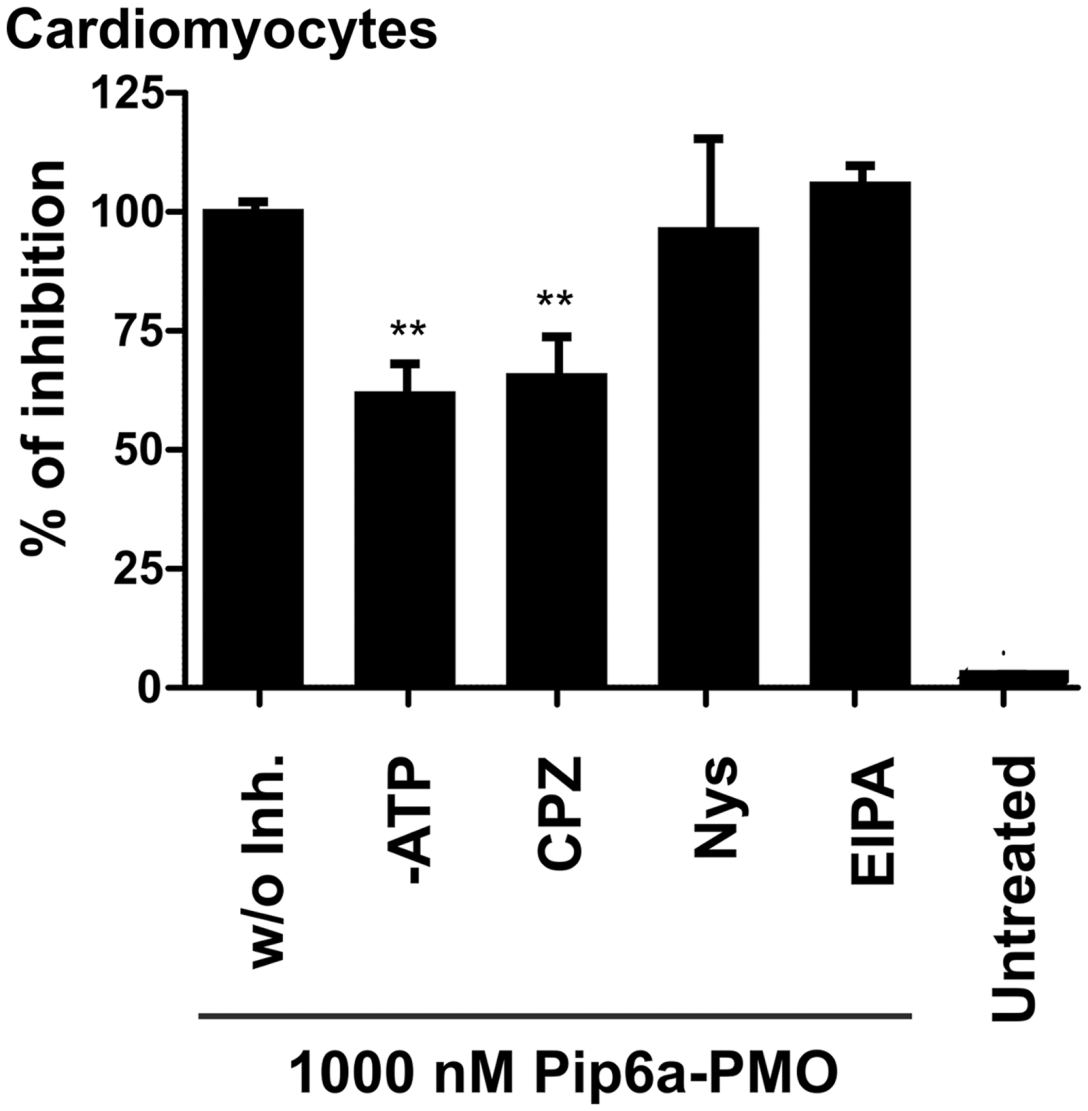
Effect of endocytosis inhibitors on Pip6a-PMO uptake at 4 h post-treatment. Effect of energy depletion and endocytosis inhibitors on the uptake of Pip6a-PMO (at 1000 nM) in wild-type cardiomyocytes at 4 h post-treatment as measured by fluorescence spectroscopy. The following inhibitors were used at the indicated concentrations: NaN_3_ (10 mM) and 2′-Deoxy-D-Glucose (DDG) (6 mM) for ATP-depletion (-ATP); chlorpromazine (CPZ, 15 µM) for clathrin-mediated endocytosis inhibition; nystatin (Nys, 25 µM) for caveolae-mediated endocytosis inhibition; and 5-(*N*-ethyl-*N*-isopropyl) amilorid (EIPA, 10 µM) for macropinocytosis inhibition (*n* ≥ 4).

To assess whether Pip6a-PMO is internalized by the same pathway in cardiomyocytes as in H2k *mdx* myotubes, cellular trafficking was monitored at a 1000 nM Pip6a-PMO concentration by fluorescence spectrometry in the presence of various endocytosis inhibitors. As in the case of *mdx* myotubes, ATP depletion had a negative effect on the uptake (Figure 6), indicating the involvement of an energy-dependent mechanism. Interestingly, uptake of Pip6a-PMO was inhibited by CPZ (Figure 6), an inhibitor of clathrin-mediated endocytosis, suggesting that different pathways are used for cell entry into cardiomyocytes as compared with skeletal muscle cells (Figure 4).

Collectively these results indicate that also in the cell culture models exon skipping is much less efficient in the heart muscle cells than in the skeletal muscle cells, which is in a perfect agreement with *in vivo* data, and one possible explanation for this could be that Pip6a-PMO is taken up and traffics via different endocytic routes in these two different muscle cells types.

## DISCUSSION

Covalent conjugation of CPPs to PMOs (PPMOs) has shown great promise in pre-clinical models of DMD (18). Pip6a-PMO in particular promoted efficient exon skipping and dystrophin restoration in skeletal muscles and, importantly, was the most active PPMO in cardiac muscle when injected intravenously into *mdx* mice (28). Although further studies are in progress to evaluate various *in vivo* parameters for Pip6a-PMO, little is known concerning the cellular internalization mechanism of Pip6a-PMO and the reasons why it has lower efficiency in cardiac muscles as compared with other muscle types. We, therefore, compared its behaviour in skeletal and heart muscle cellular models both with DMD and normal phenotypes.

The exon-skipping activity of Pip6a-PMO in skeletal H2k *mdx* myotubes is remarkably efficient at low concentrations, reaching a half-maximal effect (EC_50_) at 41 nM (Figure 1A and C), in line with previous data (28). Pip6a-PMO was much more effective in exon skipping than naked PMO and previously described PPMO conjugates. For example, reported exon-skipping levels were ∼33% for (RXR)_4_-PMO (21) and ∼45% for B-PMO (22) at 1 µM concentration, whereas complete exon skipping was achieved at this concentration with Pip6a-PMO (∼70% at 125 nM). A full comparison of EC_50_ values is difficult since complete dose-response curves have usually not been reported.

One major drawback that is frequently associated with CPP-based vectors is their sensitivity to the presence of serum (33,34) and the effect of serum is not often documented. On the contrary, the exon-skipping activity of Pip6a-PMO is even slightly increased (EC_50_ = 30 nM) in the presence of serum proteins (Figure 1B and C). This was perhaps not unexpected, as Pip peptides have been designed to resist degradation by proteases/peptidases (26–28).

DMD-affected muscles have been reported to be ‘leaky’ due to increased sarcolemma membrane permeability arising from the lack of dystrophin (30). Leakiness is also thought to be responsible for the improved uptake of naked SSOs, but little direct evidence has been provided. We, therefore, studied the behaviour of Pip6a-PMO conjugate and naked PMO in differentiated myotubes with a DMD (i.e. H2k *mdx* cells) or a normal (C2C12 cells) phenotype. Our results indicate that Pip6a-PMO is taken up into both types of myotubes with comparable efficiency and, accordingly, the parity is retained in exon-skipping levels (Figure 2). Moreover, the uptake and intracellular distribution of Pip6a-PMO follow a similar pattern in H2k *mdx* and C2C12 myotubes (Figure 3 and Supplementary Figure S5C). Slight differences in the biological activity of Pip6a-PMO could be detected, however at longer incubation time, i.e. 24 h, exon-skipping activity in the H2k *mdx* myotubes (EC_50_ = 30 nM) was slightly higher than in C2C12 myotubes (EC_50_ = 107 nM) (Supplementary Figure S3). In contrast, naked PMOs were not taken up and did not promote any significant exon skipping at comparable concentrations (up to 1 µM).

Collectively, our data support the idea that cell ‘leakiness’ associated with a lack of dystrophin does not have a profound effect on the exon-skipping efficiency of Pip6a-PMO in differentiated skeletal muscle cells and does not promote the uptake of naked PMO at least in cell culture. However, conclusions should be carefully drawn, as cells in culture are not stressed by physical forces as occurring in a flexing muscle.

It has been reported that dystrophin restoration by SSOs in different groups of skeletal muscles is not uniform, with higher activity achieved in certain muscles such as *tibiaIis anterior* (28). In this context, we hypothesized that different SSO/PMO uptake depends on the regenerative capacity of the various muscle types (i.e. different activity or proportion of myoblasts). Therefore, cellular uptake/trafficking of SSOs/PPMOs in undifferentiated and differentiated cells could be different. Our data show that the internalization of Pip6a-PMO is significantly lower in myoblasts than in myotubes (both in H2k *mdx* or C2C12 cells) (Supplementary Figure S5C). Moreover, the intracellular trafficking of Pip6a-PMO is completely different in myoblasts, where Pip6a-PMO is mostly confined in endocytic vesicles, whereas a more diffuse cytoplasmic distribution and a strong nuclear accumulation are prevalent in myotubes (Figure 3 and Supplementary Figure S5C). However, this is not due to different densities of nuclei, as equal amounts were found in myoblasts and myotubes (data not shown). It thus seems that endosomal entrapment of Pip6a-PMO, which is much higher in myoblasts than in myotubes, contributes more to the lower exon-skipping efficiency in myoblasts as compared with myotubes (EC_50_ = 183 nM in H2k *mdx* myoblasts *versus* EC_50_ = 30 nM in myotubes) than the slightly lower cellular uptake (Figure 2).

Regarding cardiac muscle, on systemic injections of *mdx* mice, Pip6a-PMO is remarkable in promoting dystrophin expression in the heart, although less efficiently than in skeletal muscles (28). The reason for this has not been clear. Recently, Pip5e-PMO (from which Pip6a-PMO was derived) showed increased nuclear accumulation in the cardiac muscle tissue in live heart slices compared with B-PMO (27). It is worth noting that Pip5e and Pip6a differ only by inversion of the central 5 amino acid core and otherwise show identical exon-skipping levels in *mdx* skeletal muscle cells (27, 28). An initial report using RXR_4_-PMO has shown 22% exon 23 skipping in isolated *mdx* mouse cardiomyocytes, apparently similar to that in H2k *mdx* cells (36), but no direct comparison of a Pip-PMO in cardiac muscle cells has been reported before. Our study of Pip6a-PMO in primary cardiomyocytes derived from neonatal *mdx* and wild-type mice hearts [these cells are fully differentiated as attested by their beating properties (37)] showed that Pip6a-PMO is less efficient in inducing exon 23 skipping in cardiomyocytes than in skeletal muscle cells (with an EC_50_ ∼1 µM in cardiomyocytes as compared with EC_50_ between 30 and 200 nM in skeletal muscle cells) (Figure 5B). By contrast, no significant difference was found in the exon-skipping activity of Pip6a-PMO between DMD and wild-type phenotypes. Thus, in both skeletal and cardiac muscle cells, the DMD phenotype does not seem to be an important determinant of Pip6a-PMO efficiency. This thoroughly disproves the concept that PMOs and PPMOs are only active because *mdx* cells are somehow more ‘leaky’ than normal cells. Further, most Pip6a-PMO remains entrapped in endocytic vesicles with a pattern reminiscent of the distribution found in skeletal muscle myoblasts. Therefore, unfavourable intracellular trafficking and greater endosomal entrapment appear to be a major cause of the decreased cardiac activity of Pip6a-PMO compared with skeletal muscles.

CPPs conjugated to various cargoes are reported to internalize in cells via different endocytic pathways, and this is believed to be important for their intracellular trafficking and ultimate fate inside the cells (33,35). In an earlier study, it was shown in the HeLa pluc705 cell line (a widely used model to study SSOs trafficking) that Pip2b-PNA conjugates used clathrin-mediated endocytosis to gain access to the cells (26). However, studies carried out in a cancer cell line cannot be extrapolated to muscle cells. In this study, we aimed to elucidate which endocytic pathways would be responsible for the trafficking and biological activity of Pip6a-PMO in muscle cells. For this purpose, we investigated the impact of different pharmacological endoytosis inhibitors (used at highest non-toxic concentration in a given cell type) on the Pip6a-PMO-mediated delivery to the different muscle cell types, focusing on skeletal and cardiac muscle cells. In H2k *mdx* cells, Pip6a-PMO is trafficking by an energy-dependent process involving caveolae-mediated endocytosis (Figure 4 and Supplementary Figure S6). Interestingly, the same trend was observed in both undifferentiated myoblasts and differentiated myotubes, indicating that the lower exon-skipping activity of Pip6a-PMO (Figures 1 and 2) in myoblasts is not due to differences in the uptake pathway (Figure 4C). Moreover, we showed that caveolae-mediated uptake mechanism also occurs in C2C12 cells (data not shown), which confirms that this endocytosis route is the preferential one in skeletal muscle cells with both DMD and wild-type phenotypes.

As found in H2k *mdx* cells, Pip6a-PMO trafficking is energy-dependent in primary cardiomyocytes (Figure 6). Intriguingly, in contrast with the skeletal muscle cells, Pip6a-PMO uptake in cardiomyocytes occurs mainly through clathrin-mediated endocytosis. This suggests that the caveolae-mediated pathway is more favourable to promote the delivery of Pip6a-PMO to the nuclear compartment and could provide an explanation for the higher exon-skipping activity in skeletal as opposed to cardiac muscles.

In conclusion, we have comprehensively characterized the cellular trafficking of Pip6a-PMO in skeletal and cardiac muscle models of DMD. First, nuclear delivery is more efficient in differentiated myotubes than in undifferentiated myoblasts in skeletal muscle cells. Second, cardiomyocytes behave differently from H2k *mdx* myotubes both in terms of Pip6a-PMO uptake pathway and in terms of cellular trafficking. In both cases, segregation within endocytic vesicles seems to limit nuclear delivery and as a consequence exon-skipping efficiency. Cell screening in primary cardiomyocytes isolated from *mdx* mice in addition to H2k *mdx* skeletal muscle cells before *in vivo* evaluation would be predicted to accelerate the discovery of new and more efficient PPMOs and lead to clinically promising developments.

## SUPPLEMENTARY DATA

Supplementary Data are available at NAR Online.

## FUNDING

Association Française contre les Myopathies (AFM), [14784 to M.J.G., M.J.A.W. and B.L.]; The work in the laboratory of M.J.G. was also supported by the Medical Research Council (MRC) [U105178803] and in the laboratory of M.J.A.W. by the Medical Research Council [G0900887]; Work in the laboratory of B.L. was partly funded by the Centre National de la Recherche Scientifique (CNRS); The EU-Lifelong Learning Programme (ERASMUS Placements) supported (to S.G.). Funding for open access charge: Association Française contre les Myopathies (AFM), [14784].

*Conflict of interest statement*. None declared.

## Supplementary Material

Supplementary Data
