# Supplementary material for: Cellular trafficking determines the exon skipping activity of Pip6a-PMO in mdx skeletal and cardiac muscle cells
Source: Nucleic Acids Res. 2013 Dec 22;42(5):3207–17. doi: 10.1093/nar/gkt1220 (PMC3950666; doi:10.1093/nar/gkt1220)
Supplement: Supplementary Data [file supp_42_5_3207__index.html]

Cellular trafficking determines the exon skipping activity of Pip6a-PMO in mdx skeletal and cardiac muscle cells — Cellular trafficking determines the exon skipping activity of Pip6a-PMO in mdx skeletal and cardiac muscle cells — Supplementary Data 

# Cellular trafficking determines the exon skipping activity of Pip6a-PMO in *mdx* skeletal and cardiac muscle cells

## Supplementary Data

files

**Files in this Data Supplement:**

- Supplementary Data - doc file
