# Supplementary material for: Cellular trafficking determines the exon skipping activity of Pip6a-PMO in mdx skeletal and cardiac muscle cells
Source: Nucleic Acids Res. 2013 Dec 22;42(5):3207–17. doi: 10.1093/nar/gkt1220 (PMC3950666; doi:10.1093/nar/gkt1220)
Supplement: Supplementary Data [file supp_gkt1220_nar-02179-y-2013-File008.doc]

**Supplementary Information**


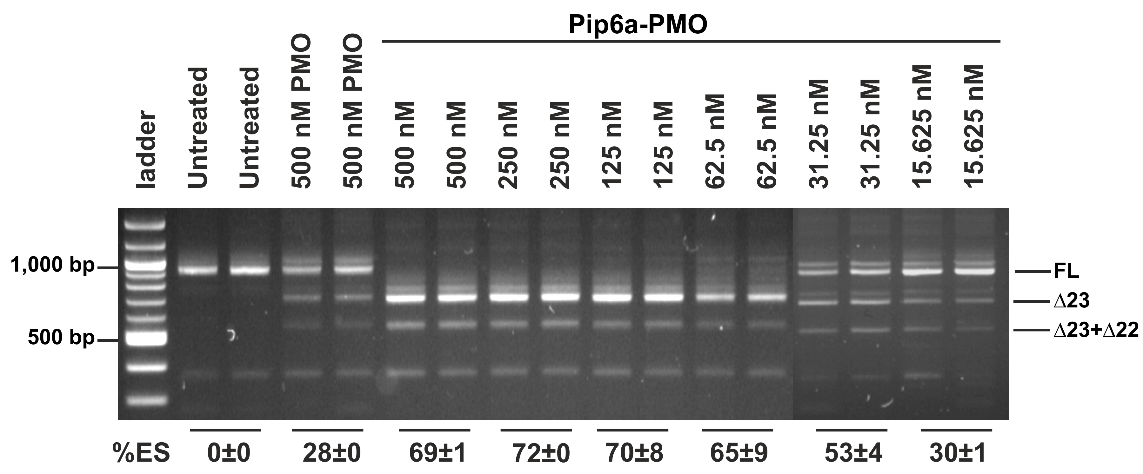


**Figure S1. Exon skipping efficiency in H2k *mdx* myotubes at 48 h post-treatment.**

H2k *mdx* cells were differentiated for 5 days and thereafter incubated with Pip6a-PMO under serum condition (+S). Transfections were carried out for 4 h under serum-free or serum condition and cells were incubated further with serum-containing medium for 44 h. Exon skipping efficiency was evaluated on pre-mRNA levels by nested RT-PCR. Products were separated by gel electrophoresis and exon skipping values were derived from densitometric analysis. Percentage of exon skipping (%ES) is calculated only based on the densitometric value of the Δ23 band related to the other bands (full length + Δ23 + Δ23+22) with n≥4. An EC_50_ value of 20 nM was calculated using Prism 5.0 software after normalization of the Δ23 skipping.


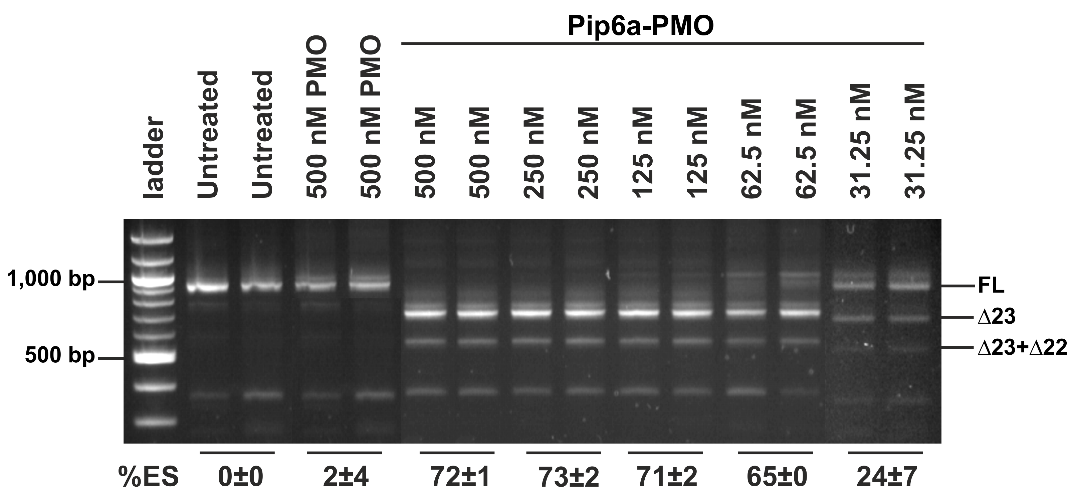


**Figure S2. Exon skipping with unlabelled Pip6a-PMO conjugate in H2k *mdx* myotubes at 24 h post treatment.**

H2k *mdx* cells were differentiated for 5 days and thereafter incubated with Pip6a-PMO under serum condition (+S). Transfections were carried out for 4 h under serum-free or serum conditions and cells were incubated further with serum-containing medium for 20 h. Exon skipping efficiency was evaluated on pre-mRNA levels by nested RT-PCR. Products were separated by gel electrophoresis and exon skipping values were derived from densitometric analysis. (Percentage of exon skipping (%ES) calculated as described in Figure S1 with n≥4).

**
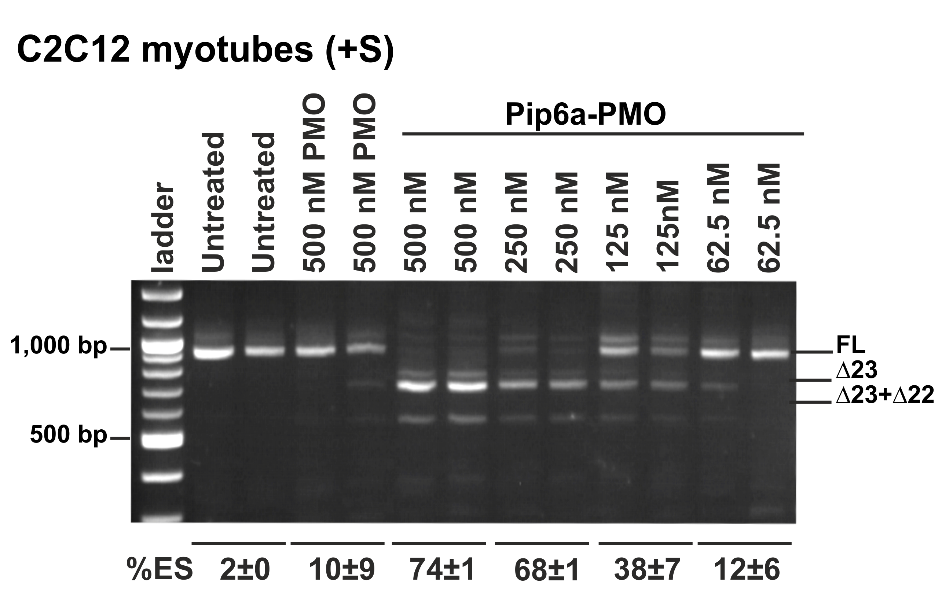
**

**Figure S3. Exon skipping efficiency of Pip6a-PMO in C2C12 skeletal muscle cells.** C2C12 cells were differentiated for 6 days and thereafter incubated with Pip6a-PMO under serum condition (+S). Transfections were carried out for 4 h under serum conditions and cells were incubated further with serum-containing medium for 20 h. Exon skipping efficiency was evaluated on pre-mRNA levels by nested RT-PCR. Products were separated by gel electrophoresis and exon skipping values were derived from densitometric analysis. (Percentage of exon skipping (%ES) calculated as described in Figure S1 with n≥4).

**
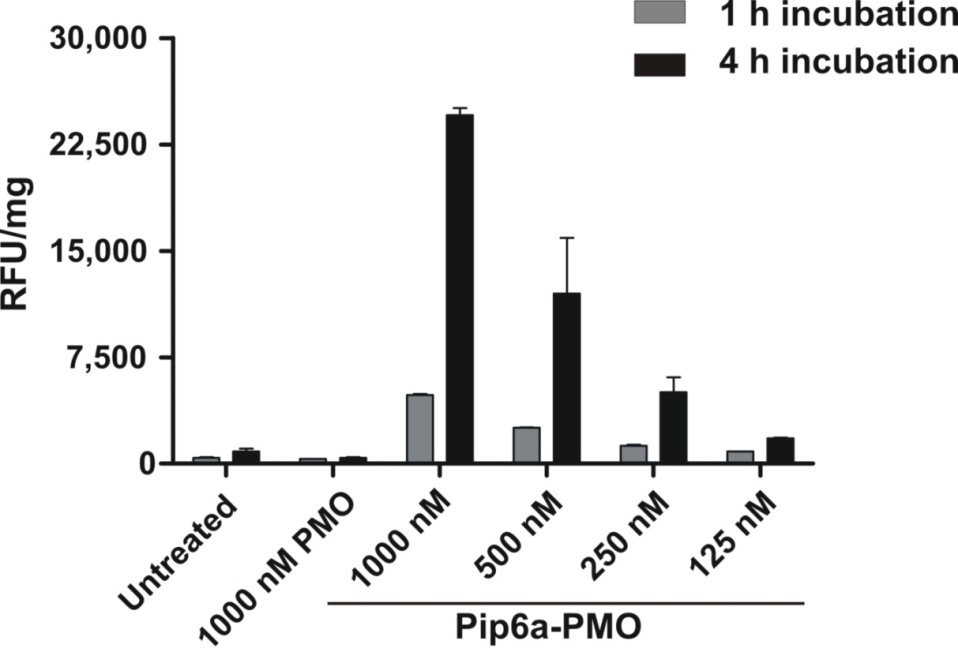
**

**Figure S4. Determination of the incubation time for uptake measurement.** Differentiated H2k *mdx* cells were incubated 1h or 4h at the indicated concentration with Pip6a-PMO. The dose-depended internalization was accessed by fluorescence spectroscopy (n≥2).

***
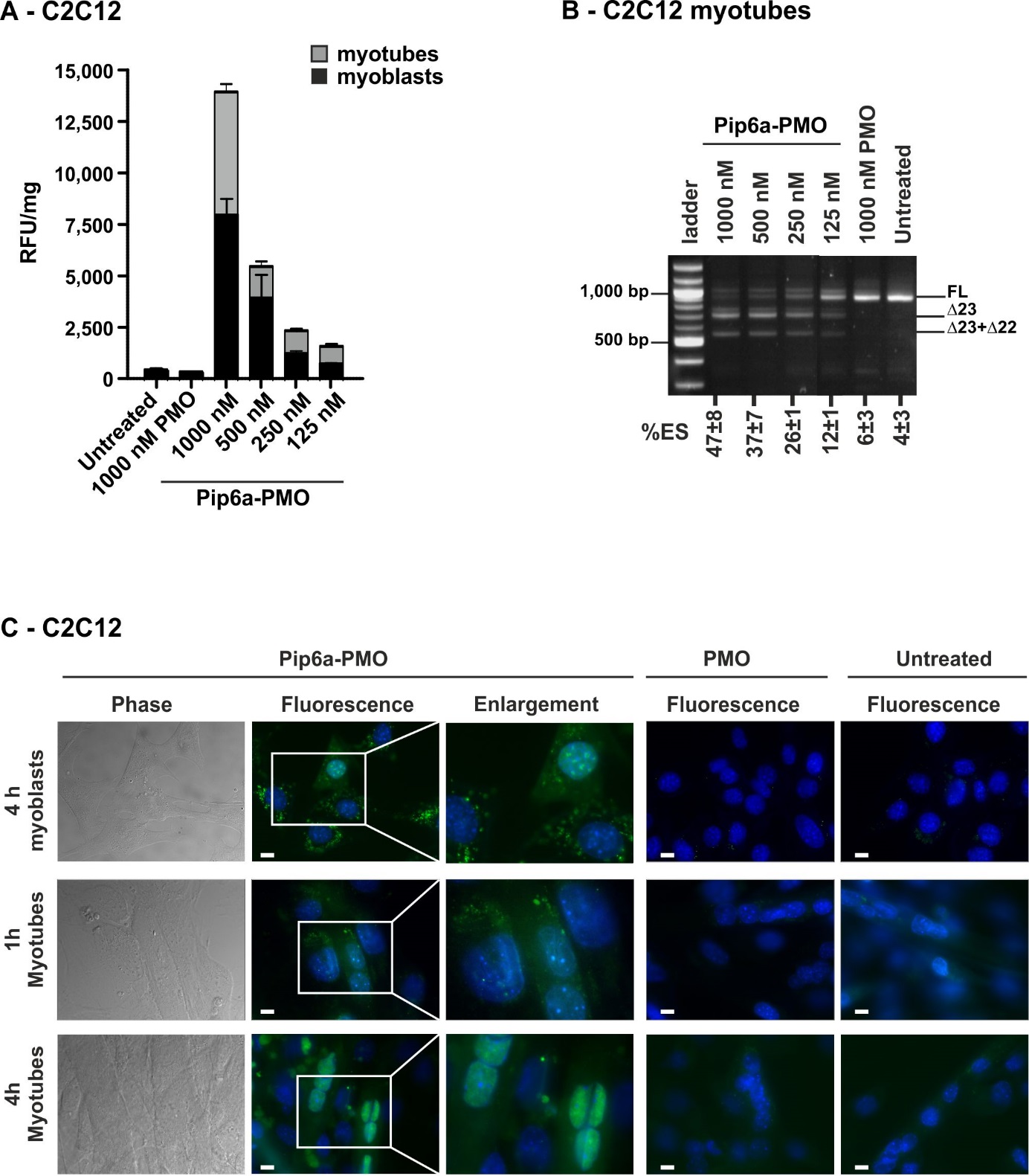
***

**Figure S5. Comparative analysis of Pip6a-PMO uptake and exon skipping in C2C12 skeletal muscle cells.** Differentiated and non-differentiated C2C12 cells were incubated 4h at the indicated concentration with Pip6a-PMO. The dose-depended internalization was accessed by fluorescence spectroscopy (A). Using the same differentiated C2C12 samples, exon skipping efficiency cells was evaluated by RT-PCR (B). (Percentage of exon skipping (%ES) is calculated as described in Figure S1 with n≥4).

(C) Representative images of C2C12 myoblasts or myotubes which were incubated with fluorescein-labelled Pip6a-PMO (1000 nM) or PMO (1000 nM) for the indicated times. Cell nuclei were labelled with Hoechst dye. Untreated cells are shown as controls. Pip6a-PMO or naked PMO distribution in live unfixed cells was evaluated by fluorescence microscopy with a Zeiss Axiovert 200 M. White bar = 10 µm.


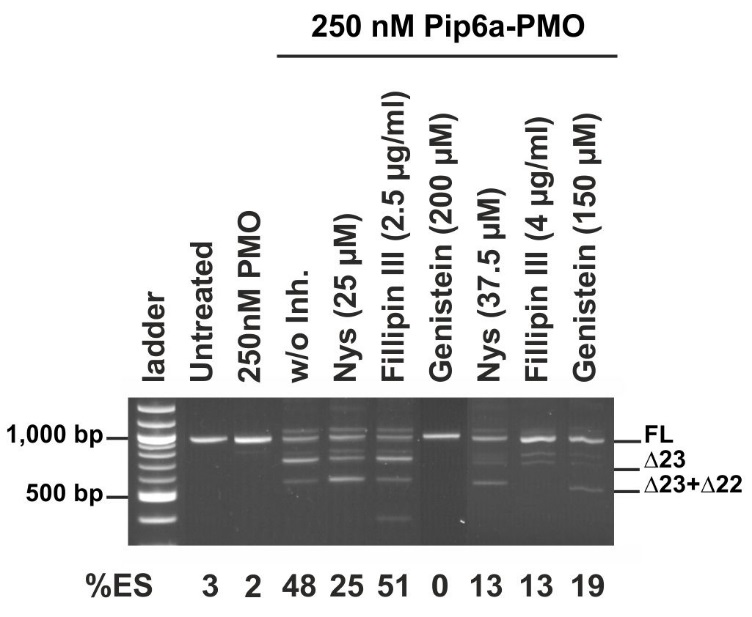


**Figure S6. Inhibition of caveolae-mediated endocytosis with Filipin III and Genistein in H2k *mdx* myotubes.**

Effect of caveolae-dependant endocytosis inhibitors on the uptake of Pip6a-PMO (at 250 nM) in H2k *mdx* myoblasts at 4 h post-treatment on exon skipping. Treatment with inhibitors was started 30 min prior to the treatment with Pip6a-PMO. The following inhibitors were used at the indicated concentrations: Nystatin (Nys, 25 µM and 37.5 µM), Flillipin III (2.5 µg/ml and 4 µg/ml) and Genistein (200 µM and 150 µM). (Percentage of exon skipping (%ES) calculated as described in Figure S1 with n=2).

**
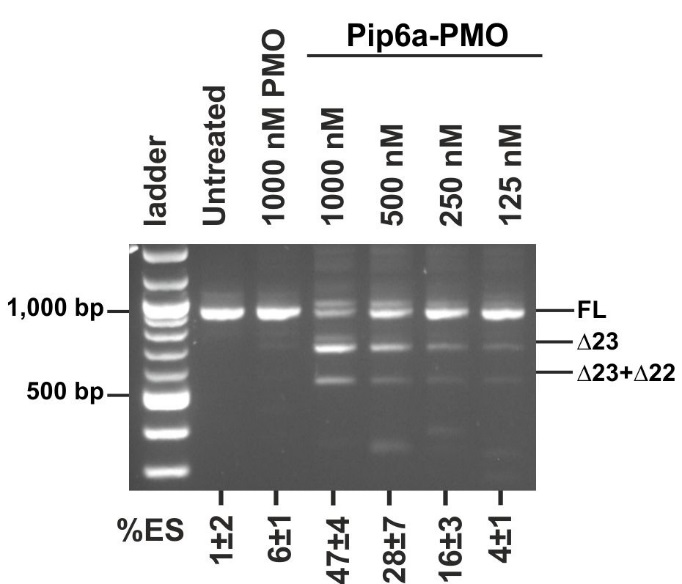
**

**Figure S7. Exon skipping efficiency in primary wild type cardiomyocytes at 48 h post-treatment.**

Exon skipping efficiency of Pip6a-PMO at 48 h post transfection in primary wild type cardiomyocytes.

RT-PCR analyses and percentage of exon skipping (%ES) as described in Figure S1 with n≥4.
